# Development of bismuth sulfide nanorods and polyamidoamine dendrimer on reduced graphene oxide as electrode nanomaterials for electrochemical determination of salbutamol

**DOI:** 10.1038/s41598-023-36028-0

**Published:** 2023-06-01

**Authors:** Mahshid Padash, Shahab Maghsoudi, Mehdi Mousavi

**Affiliations:** 1 Department of Chemistry, Shahid Bahonar University of Kerman, P.O. Box 76175-133, Kerman, Iran; 2 Young Researchers Society, Shahid Bahonar University of Kerman, P.O. Box 76175-133, Kerman, Iran

**Keywords:** Environmental sciences, Health care, Chemistry, Materials science, Nanoscience and technology

## Abstract

Dendrimers, a new class of nanomaterials, are receiving more attention in various fields. In this study, by combining the advantages of polyamidoamine (PAMAM) dendrimer with reduced graphene oxide (rGO) and bismuth sulfide (Bi_2_S_3_), we came to design a new composite and its application for electrochemical sensors was investigated for the first time. As a new approach in the preparation of the composite, PAMAM was used for the first time to increase the surface of Bi_2_S_3_ with rGO, which ultimately led to an increase in the active surface area of the sensor (5 times compared to the bare electrode). For the first time, we used the sonochemical method for interaction between PAMAM with Bi_2_S_3_ and rGO, which was a simpler and faster method to prepare the composite. The purposeful design of the composite was done by using the experimental design method to obtain the optimum composition of components. The new nanocomposite was successfully applied for simple and sensitive electrochemical sensing of salbutamol for controlling the health of food. Salbutamol is used as a prohibited additive in animal and poultry feed. The sensor has good sensitivity (35 times increase compared to the bare electrode) and a low detection limit (1.62 nmol/L). Moreover, it has acceptable selectivity, good repeatability (1.52–3.50%), good reproducibility (1.88%), and satisfactory accuracy (recoveries: 84.6–97.8%). An outstanding feature of the sensor is its broad linear range (5.00–6.00 × 10^2^ nmol/L). This sensor is well suited for the determination of salbutamol in milk, sausage, and livestock and poultry feed samples.

## Introduction

Salbutamol (SAL) is a kind of β2-adrenergic agonists with an aromatic ring and a terminal amino group. Salbutamol can help to raise animal growth and feeding efficiency by decreasing body fat and increasing protein accretion^1^. However, it can stack in animals and can be easily accumulated in human tissues after meat consumption, which can lead to health-related issues^2,3^. To protect public health, the Food and Agriculture Organization (FAO), European Union (EU), and China have declared that the β2-adrenergic agonists, including salbutamol, must be zero in animal food intake^4^. However, illegal abuse of salbutamol in animal feed never stops. Consequently, it is necessary to spread a simple, fast, and sensitive method for screening salbutamol at low concentrations in feed and food samples for food safety control.

Several methods have been reported for detecting SAL, including high-performance liquid chromatography^5^, immunochromatography^6^, liquid chromatography-mass spectrometry^7^, and electrochemical methods. The electroanalytical methods have simple pre-treatment procedures, low cost, high sensitivity, short analysis time, and miniaturizable instrumentation. Therefore, electroanalytical methods have gained great attention, especially in routine inspection. Various electrochemical methods based on cyclic voltammetry (CV)^8^, differential pulse voltammetry (DPV)^9^, linear sweep voltammetry (LSV)^10^, electrochemical impedance spectroscopy (EIS)^11^, and amperometry^12^ have been developed for determinations of salbutamol. Thus, the design and development of a selective and sensitive sensor for SAL determination through electroanalytical technique gained significant interest in scientific, medical, and health communities.

Novel advanced material development with versatile applications is still a challenge for the scientific community. The design of developed materials plays an important role in improving the performance of electrochemical sensors. Dendrimers are a new class of nanomaterials that are repetitively globular branched molecules with three-dimensional structure, terminal functional groups, and well-defined cavities which can act as hosts for other molecules^13^. Among various dendrimers, the most widely used dendrimer is poly(amidoamine) (PAMAM)^14^. Polyamidoamine (PAMAM) dendrimers are “dense star” polymers that have 11 different generations with ten functional surface groups. With a series of repetitive groups, each new generation of PAMAM is formed around the preceding generation. The newly formed PAMAM has outstanding properties. These properties are a large diameter, ample surface, and they possess more reactive branches. The reactive surface branches allow PAMAM to be considered as affinity ligands and detecting agents for pharmaceutical compounds^15^. They have received great attention in design and development of electrochemical sensors, due to their advantages, such as stable molecular weight, molecular uniformity, specific size, definite shape, and lots of surface branches^16^. Among common candidates for fabrication of high performance dendrimer-based electrochemical sensors, is combining dendrimers with many conductive materials.

Today, various conductive materials are used to increase the sensitivity and selectivity of electrochemical sensors. Some well-known modifiers which have a significant impact on the performance of sensors, e.g., Afzali et al. used multi-walled carbon nanotubes (MWCNTs) to design a sensitive sensor for the detection of zearalenone and antimony (III)^17,18^, and Ostovar et al. also applied MWCNT/Poly pyrrole/phenylboronic acid for electrochemical measurement of diltiazem^19^.

Bismuth sulfide (Bi_2_S_3_) is an n-type semiconductor with a direct energy band gap ranging from 1.2 to 1.7 eV. The n-type semiconductor material contains a large number of free electrons, which play a significant role in electrical conductivity. Bi_2_S_3_ is known as a powerful sensor modifier because of its excellent photovoltaic features, natural abundance, and desirable environmental compatibility^20^. It can be combined with carbon substrate to prevent agglomeration, which leads to a decline in the effective surface area^21^.

Two-dimensional (2D) nanomaterials with layered structures, high surface area, mechanical flexibility, and electronic properties have attracted much attention in the design of sensors. Among the most widely used two-dimensional nanomaterials, we can mention graphene and its derivatives (graphene oxide reduced graphene oxide(. In recent years, a new family of two-dimensional materials, transition metal carbides/nitrides (MXene), has received a lot of attention. Despite its advantages, MXene still has its own challenges and disadvantages. Two major limitations in the preparation of MXene based materials are poor water dispersibility of MXene and rapid oxidation of the MXene. Therefore, this material is still in its early stages and needs more research to improve it^22,23^. Instead, two-dimensional graphene nanomaterials and its derivatives, in addition to good electrical properties and high active surface, have good stability and dispersion in water. The preparation of these nanomaterials is also cost-effective and does not require advanced equipment.

Graphene oxide (GO) is a two-dimensional one-atom-thick sp2 -bonded carbon network^24^. The electrochemical properties of GO, such as fine electron transport and great electrocatalytic activity, have resulted in a wide-range of applications as an electrode material^25^. To enhance the electrochemical activity of GO, it is transformed to reduced graphene oxide (rGO) by removing almost all the oxygen-containing groups^26^. Reduced graphene oxide (rGO) has attracted great attention in electrochemical sensors^27^.

The design of a sensor with fast, simple, and efficient screening of salbutamol can be widely used in food quality control centers. A suitable sensor for determination of salbutamol in animal and poultry feed and food samples should, in addition to proper performance, be easy, fast and economical to prepare in order to be able to be commercialized. Therefore, in the design of the modifier for the preparation of the sensor, both the aspect of improving the performance of the sensor and the aspect of its commercialization were considered. The biocompatibility of the materials used to produce the sensor is also of particular importance. Joan Chepkoech Kilel and colleagues used MWCNT to prepare a sensor for salbutamol determination. Despite its benefits, MWCNT is considered to be hazardous, its preparation technology is expensive, and its length and geometric structure can limit some applications^28^. Wang and his colleagues used silver-palladium nanoparticles to prepare the sensor. Both of these metals are expensive and their use is not economical^4^. Therefore, we tried to use materials that, in addition to improving the performance of the sensor, are biodegradable and inexpensive materials that are easy to prepare and do not require expensive equipment.

Herein, we developed a new dendrimer-based nanocomposite for electrochemical sensing of SAL in food samples. PAMAM was selected as a representation of dendrimer. Bi_2_S_3_ and rGO were used for improvement of conductivity of dendrimer (as conductive materials). The experimental design (central composite design (CCD) and response surface methodology) method was implemented to understand curvature and interaction terms and optimize the experimental variables (factors) affecting the performance of nanocomposite. Finally, rGO/ PAMAM /Bi_2_S_3_ nanocomposite was fully characterized and the fabricated sensor was evaluated and applied for SAL determination in real samples.

## Results and discussion

### Characterization of synthesized rGO, Bi_2_S_3_, and PAMAM

**Figure 1:**
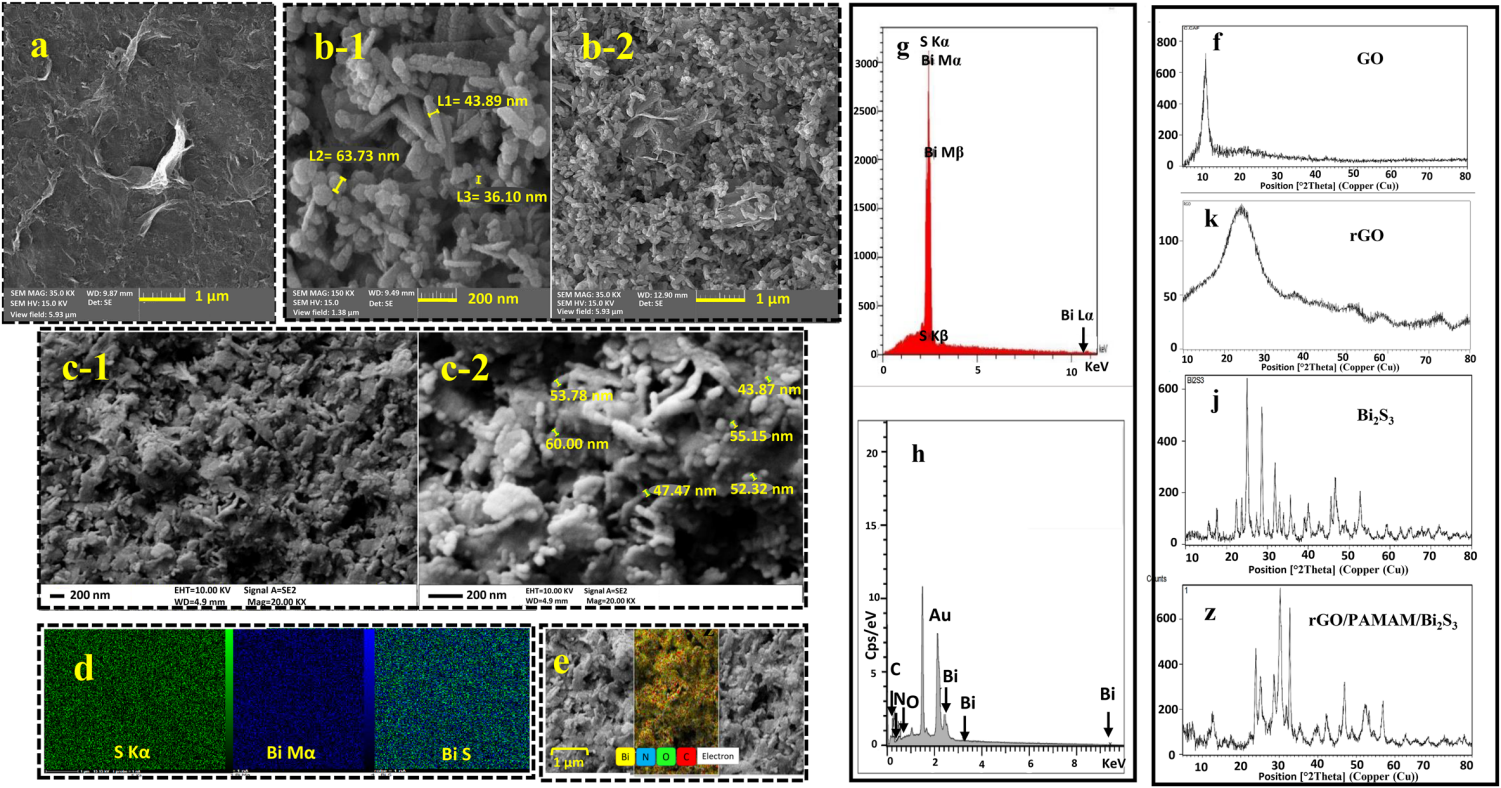
FE-SEM images: (**a**) rGO, (**b-1, b-2**) Bi_2_S_3_, (**c-1, c-2**) rGO/PAMAM/Bi_2_S_3_, mapping: (**d**) Bi_2_S_3_, (**e**) rGO/PAMAM/Bi_2_S_3_, and EDX: (**g**) Bi_2_S_3_, and (**h**) rGO/PAMAM/Bi_2_S_3_. XRD analysis of (**f**) GO, (**k**) rGO, (**j**) Bi_2_S_3_, (**z**) rGO/PAMAM/Bi_2_S_3_.

The synthesized Bi_2_S_3_, rGO, and PAMAM were characterized by field emission scanning electron microscopes (FE-SEM), energy-dispersive X-ray spectrometry (EDX), X-ray diffraction (XRD), and Fourier transform infrared (FTIR) spectroscopy. The morphology of the synthesized Bi_2_S_3_ nanorods and rGO were investigated by FE-SEM. Figure 1a–c show three-dimensional structures of the rGO, Bi_2_S_3_ nanorods, and rGO/PAMAM/Bi_2_S_3_; also, it shows specific surface area changes (increasing) during modification. EDX analysis (Fig. 1g,h) helps in assessment of the purity for the synthesized nanoparticles. Figure 1g confirms that the prepared Bi_2_S_3_ sample consisted of S and Bi elements. The elements map shows uniform distribution of contributing elements in the synthesized nanoparticles (Fig. 1d,e). The rGO sheets morphology is shown in Fig. 1a,c. As it is indicated, rGO has sheets (Fig. 1c) that the Bi_2_S_3_ nanorods are accumulated around them. The obvious thickness increase of rGO sheets (Fig. 1c) is due to PAMAM application. The XRD pattern of GO (Fig. 1f) represents a sharp diffraction peak at 2θ = 10.9787°. Using the Bragg equation (2dsinθ = nλ), it is shown that the interlayer d-spacing between the GO nanosheets was 8.05 Å. This value confirms that oxygen-containing functionalization was achieved, successfully^29^. After hydrothermal reduction of the GO, the peak at 2θ = 10.9787° disappeared, and a new weak peak appeared at 23.8897°, which was related to the diffraction of rGO (Fig. 1k). The interlayer d-spacing corresponding to rGO sheets is 3.72 Å, which is less than GO sheets. These decreases indicate that, during the reduction of GO, the oxygen-containing functional groups are removed successfully. The XRD pattern of Bi_2_S_3_ well approves the synthesis of the orthorhombic phase Bi_2_S_3_ (Fig. 1j). The diffraction peaks of rGO/PAMAM/Bi_2_S_3_ (Fig. 1z) are well-matched with the generic peaks of Bi_2_S_3_ in the CPDS card (No. 17-0320) and a peak at 2θ = 23.8897° for rGO.

**Figure 2:**
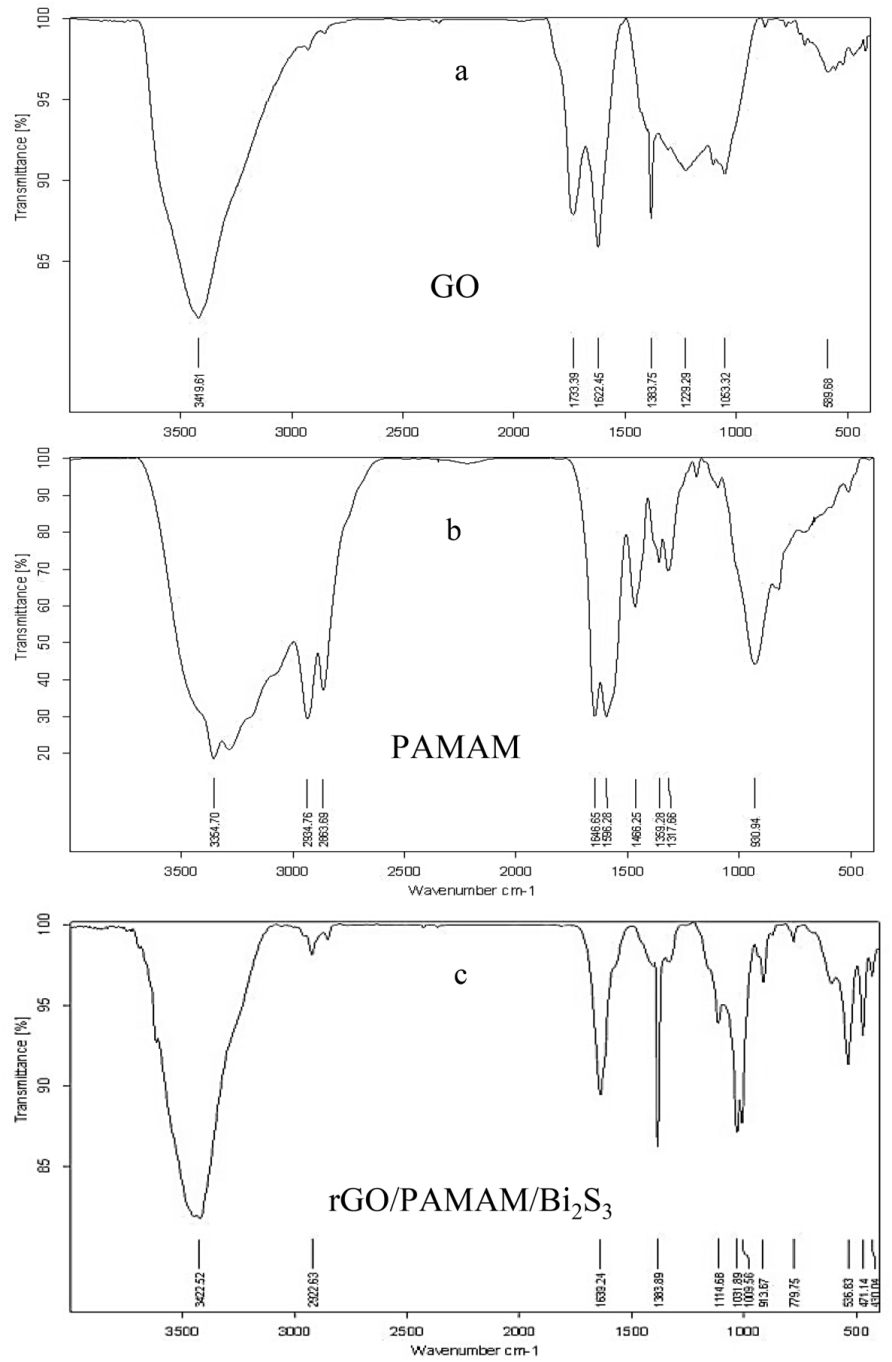
FTIR spectrums of (**a**) GO, (**b**) PAMAM, (**c**) rGO/PAMAM/Bi_2_S_3_.

To study the functional groups of GO, PAMAM, rGO/PAMAM/Bi_2_S_3_, FTIR spectroscopy was used (Fig. 2). The broad absorption band at around 3400 cm^−1^ is assigned to the stretching vibration of –OH groups. The FTIR spectra of GO confirms the presence of C=O stretching vibration peak of terminal carboxyl at 1733 cm^−1^, C=C vibrations of the aromatic ring at 1622 cm^−1^, alkoxy C–O at 1063 cm^−1^, epoxy C–O–C at 1229 cm^−1^, and tertiary C–OH at 1383 cm^−1^. These results indicate successful oxidation of graphite^30^. The band at 1114 cm^−a1^ which is assigned to the rGO/PAMAM/Bi_2_S_3_, shows the interaction between rGO and Bi_2_S_3_ nanorods^31^. In the rGO FTIR spectrum, the decreases in peak intensities of oxygen-containing functional groups indicate the efficient reduction of GO via the hydrothermal method.

**Figure 3:**
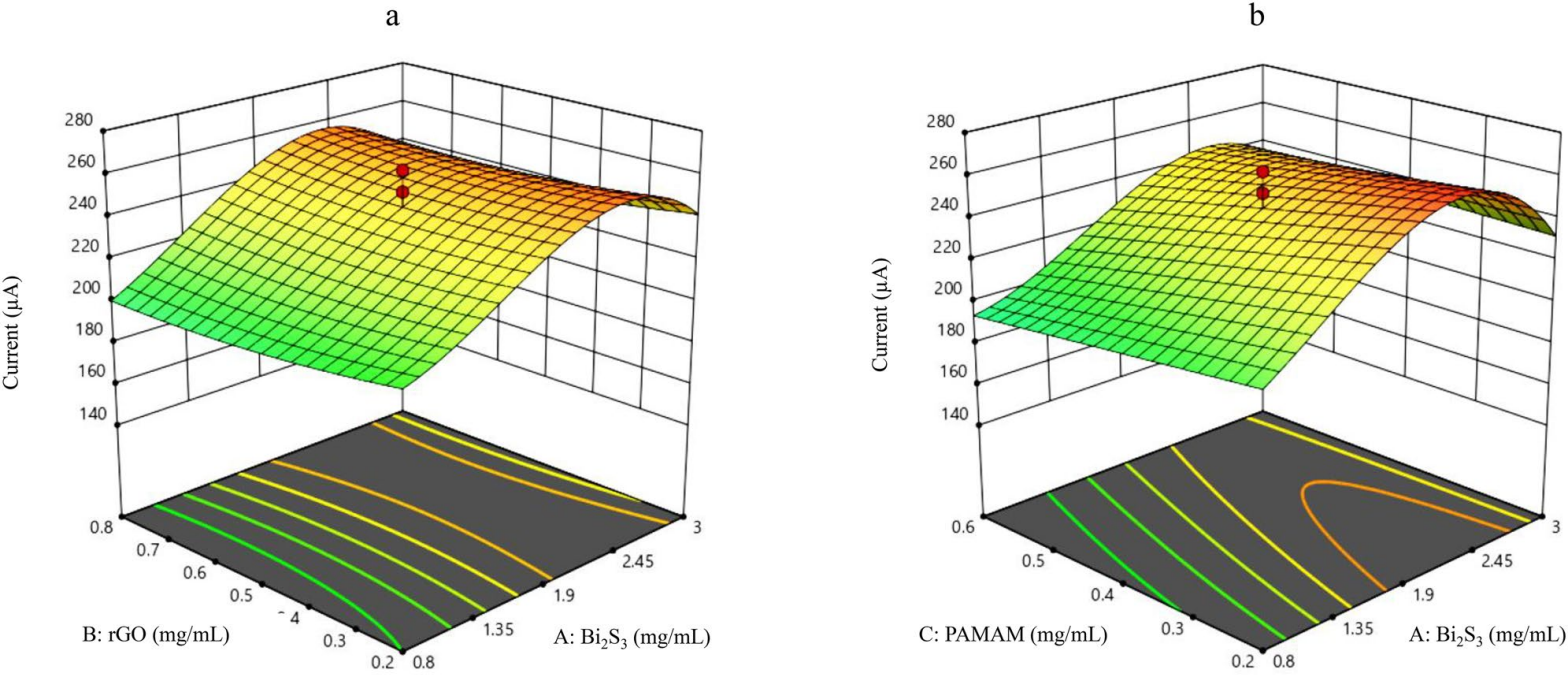
Three-dimensional display of response changes relative to factor levels using the CCD model for (**a**) rGO-Bi_2_S_3_, and (**b**) PAMAM-Bi_2_S_3_.

The FTIR spectrum of the synthesized dendrimer G3 showed absorption peaks at 3354.7 cm^−1^ for N–H stretching of primary amine and at 3270 cm^−1^ for N–H stretching of secondary amine. The aliphatic C–H stretches peaks appeared at 2934.76 cm^−1^ and 2863.69 cm^−1^. The peak at 1646.65 cm^−1^ belonged to amide carbonyl absorption, while the core N–C stretching resulted in a peak at 1596.28 cm^−1^. With the successful synthesis of the PAMAM G3 dendrimer, the peak of carboxylic carbon of C=O of aliphatic ester (1750–1735 cm^−1^) disappeared, and the absorption band related to amide carbonyl appeared at 1646.65 cm^−1^^32,33^.

### Optimization of the experimental parameters affecting in determination of salbutamol

In order to increase the sensitivity of salbutamol measurements, effective parameters related to electrochemical oxidation process were optimized using the one-at-a-time method. In the following section, optimization of the modifier preparation and the electrochemical measurement conditions are described.

### Optimization of the experimental parameters for electrode modification

The experimental design method was applied to choose the optimum composition of modifier components. To fulfill this work, central composite design and response surface modeling were used. Three affecting and interacting factors, i.e., Bi_2_S_3_, rGO, and PAMAM amounts, were considered according to our previous experience and knowledge in this field. Table S1 shows coded and uncoded levels above factors. To ensure orthogonality of the design, the number of star points (N_a_) and center points (N_o_) were set to 6 and 6, respectively. Hence, the total number of treatments were equal to (2f. + 2 × f + N_o_) = 20. The 20 treatments in the design matrix were randomly run, and the DPV current for a solution of 5 mmol/L [Fe(CN)_6_]^3−^/[Fe(CN)_6_]^4−^ in 0.1 mol/L KCl was considered as the response. Then the response surface model corresponding to CCD was generated. The contributing terms in the RSM were chosen according to the effectiveness of interaction and curvature terms which were present in the analysis of variance (ANOVA) table of the model. The significant terms involved in the coded response surface model are shown in Eq. 1. Using the coded equation and considering the coefficients of the factors, the relative impact of the factors can be identified.

1 $$ {\text{Ip}} = {244}.{9}0 + {35}.{\text{69A}} - {14}.0{\text{8 C}} + {4}.{\text{88AC}} - {27}.0{\text{9A}}^{2} + {3}.{\text{47 B}}^{2} - {4}.{\text{63A}}^{2} {\text{B}} + {1}0.{\text{96A}}^{2} {\text{C}} - {19}.{\text{81A}}^{3} $$

where A, B, and C are the amounts of Bi_2_S_3_, rGO, and PAMAM respectively. Statistical parameters of the RSM, including the coefficient of multiple determination (R^2^), adjusted R^2^, predicted R^2^, and F statistics values are equal to 0.929, 0.878, and 0.814, and 18.050, respectively. Also, the lack of fitness mean squares to pure experimental uncertainty mean square variances is 0.395, which is not significant at the *p* = 0.05 probability level. Evaluation of model statistics indicate that it fits the data significantly, and the model can describe the majority of experimental response changes (about 88%). In addition, by maximizing the RSM, an optimum condition with Bi_2_S_3_, rGO, and PAMAM concentrations of 2.248, 0.800, 0.200 (mg/mL), and the theoretical response of 267.099 (µA) were predicted. In order to evaluate the prediction accuracy of the model, three runs were performed in the predicted optimum condition and the average of these results was 265.200 (µA). This result was in good agreement with the predicted response derived by the model and confirmed the reliability of the optimization procedure. Some 3D response surface plots for the two variables, while the other factor is fixed at its central level, are shown in Fig. 3.

### Effect of supporting electrolyte and pH

In all voltammetric determinations, supporting electrolyte plays an important role, so the effect of its pH and composition were optimized. To fulfill this aim, at pH = 5.0 and 0.1 mol/L solutions of KCl, acetate, Britton–Robinson (BR), and phosphate buffers were tested (Figu. S1A). Since the highest peak current in the DPV experiments belonged to the phosphate buffer solution, it was selected as the best supporting electrolyte for the electrochemical measurements. Then DPV was carried out at a range of pH (4.0 to 7.0) to investigate the effect of pH on the oxidation reaction of SAL at rGO/PAMAM/Bi_2_S_3_/GCE. As shown in Fig. S1B, the largest current appeared at pH = 5.0, which was considered as the optimum pH for further studies.

### Effect of Differential pulse voltammetry parameters

Different parameters of DPV method that affected the electrochemical signal were optimized using 4.00 × 10^2^ nmol/L salbutamol solution in phosphate buffer (0.1 mol/L, pH = 5.0). These parameters include pulse amplitude, pulse duration, scan rate, condition time, and condition potential. As shown in Fig. S2A-E the optimum values for pulse amplitude, pulse duration, scan rate, conditioning time, and condition potential are 0.30 V, 5 ms, 0.03 V/s, 300 s, and + 0.50 V, respectively.

### Study of electrode modification for salbutamol determination

The relationship between anodic peak potential (Epa) and pH was investigated in order to evaluate the number of electrons involved in the electron process. As indicated in Fig. S3, there is a good linear relationship between Ep and pH with an equation of Ep = − 0.0605 pH + 1.2556 (R^2^ = 0.9869). The slope value of -0.0605 (V/pH) is close enough to the theoretical Nernstian one and indicates that the number of protons and electrons exchanged in the oxidation of SAL is the same. This finding follows previously proposed mechanisms that only one electron and one proton are involved in the electro-oxidation of SAL^34^.

**Figure 4:**
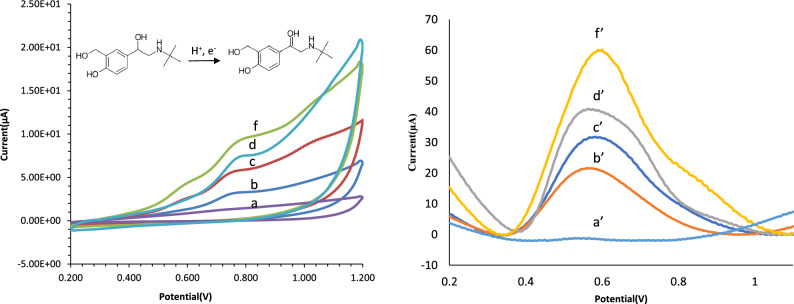
Mechanism of salbutamol oxidation, CV (scan rate: 100 mV/s) and DPV curves obtained at GCE (a, a’), GCE/Bi_2_S_3_ (b, b’), GCE/PAMAM/Bi_2_S_3_ (c, c’), GCE/rGO (d, d’), GCE/rGO/PAMAM/Bi_2_S_3_ (f, f’) in 0.1 mol/L phosphate solution (pH = 5.0) containing 2.00 × 10^2^ nmol/L salbutamol.

The electrochemical effectiveness of the modified electrode was studied using the CV and DPV method. In the voltammetry method by applying potential, the current is produced as a result of an electrochemical reaction, and the produced current is measured as a function of the applied potential. The schematic of sensing phenomena is shown in Fig. S7. To achieve this aim, the CV and DPV voltammograms of 2.00 × 10^2^ nmol/L salbutamol solution in phosphate buffer (0.1 mol/L, pH = 5.0) were tested. The oxidation peak of salbutamol results from the anodic reaction of its phenolic hydroxyl group^35^. The possible mechanism of salbutamol oxidation is presented in Fig. 4. As shown in Fig. 4, during modification of GCE, GCE/Bi_2_S_3_, GCE/rGO, GCE/PAMAM/Bi_2_S_3_ and GCE/rGO/PAMAM/Bi_2_S_3_, the signal (sensitivity) was increased significantly. These changes in sensitivity can be attributed to the higher active surface area of the modified electrode.

### Impedance analysis of the modified electrode

**Figure 5:**
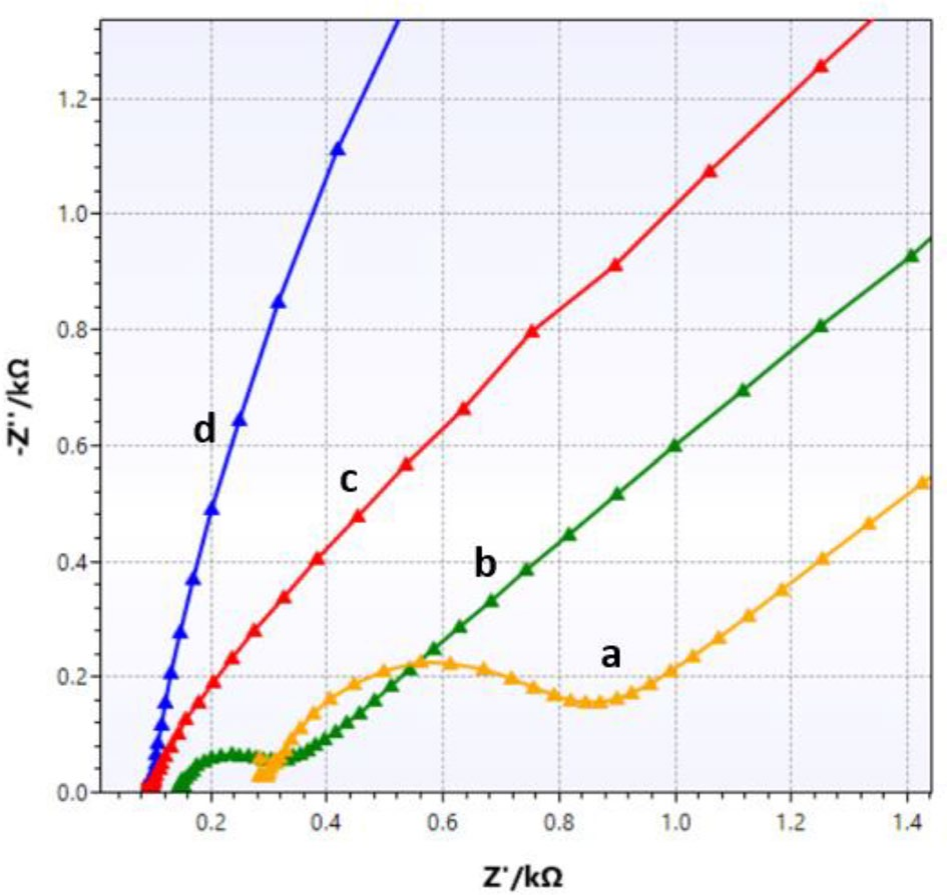
Nyquist diagrams of (**a**) bare GCE, (**b**) GCE/Bi_2_S_3_, (**c**) GCE/rGO/Bi_2_S_3_, (**d**) GCE/rGO/PAMAM/Bi_2_S_3_ in the presence of 5 mmol/L [Fe(CN)_6_]^3^/[Fe(CN)_6_]^4^ solution in 0.1 mol/L KCl. Conditions: Edc: + 0.230 V vs. Ag/AgCl; Eac: 5 mV; frequency range: 0.01–5 × 10^4^ Hz.

The electrochemical impedance spectroscopy (EIS) technique is a non-destructive method for studying the electric properties of materials. Therefore, the rate of interfacial electron transport of the modified electrode was examined by the EIS technique. Nyquist plots of bare GCE (a) GCE/Bi_2_S_3_ (b), GCE/rGO (c), and GCE/rGO/PAMAM/Bi_2_S_3_ (d) are displayed in Fig. 5. All experiments were performed using 5.0 mmol/L [Fe(CN)_6_]^3−^ and [Fe(CN)_6_]^4−^ solution in aqueous 0.1 mol/L KCl as a redox probe. In Nyquist plots, the semicircle at high frequency is related to the charge transfer resistance (Rct), and capacitance behavior is shown via the linear portion at low frequency. The equivalent series resistance (ESR) is the intercept of the semicircle with the real axis. The ESR consists of the electrolyte solution resistance, the intrinsic resistance of the active material, and the contact resistance of the interface active material and current collector. The decline in the diameter of the semicircle of the Nyquist plot after modification of the electrode indicates that the applied nanomaterials have a considerable effect on increasing the electron transfer rate. Also, the sharper slope of the linear part of the Nyquist plot of the modified electrode demonstrates improvement in the electron transfer process.

### Determination of the active surface area of the sensor

**Figure 6:**
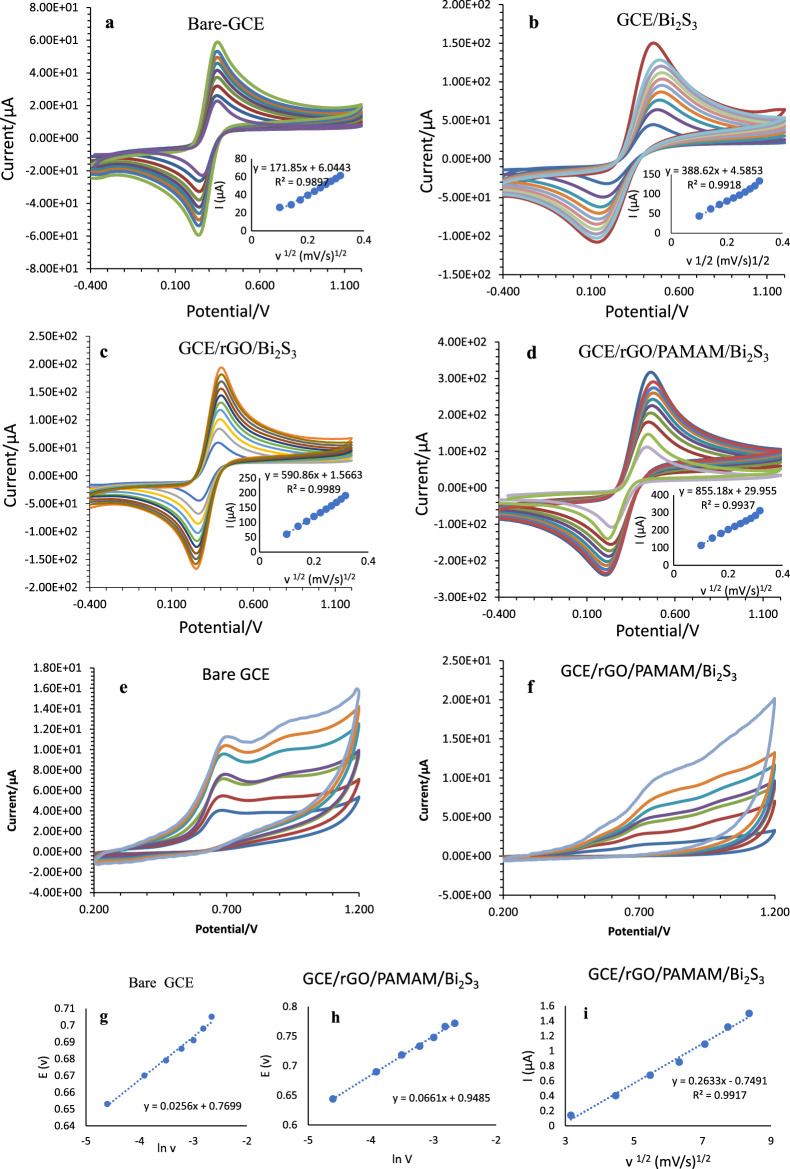
Cyclic Voltammograms of (**a**) bare GCE, (**b**) Bi_2_S_3_/GCE, (**c**) GCE/rGO/Bi_2_S_3_, and (**d**) GCE/rGO/PAMAM/Bi_2_S_3_ in 5 mmol/L [Fe(CN)_6_]^3^/[Fe(CN)_6_]^4^ solution in aqueous 0.1 mol/L KCl at a potential scan rate range of 10–100 mV/s, (**e**) bare GCE, and (**f**) GCE/rGO/PAMAM/Bi_2_S_3_ in 0.1 mol/L phosphate solution (pH = 5.0) containing 5.00 × 10^2^ µmol/L salbutamol for bare and 6.00 × 10^2^ nmol/L for modified GCE at scan rates of 10, 20, 30, 40, 50, 60, and 70 mV/s. Plots of peak potential vs. ln v (**g**) bare GCE, (**h**) GCE/rGO/PAMAM/Bi_2_S_3_. (**i**) Variation of peak currents vs. square root of scan rate for GCE/rGO/PAMAM/Bi_2_S_3_.

The active surface area of the modified electrodes was calculated using the Randles–Sevcik equation (Eq. 2).

2 $$ {\text{Ip}} = {2}.{69} \times {1}0^{{5}} \left( {{\text{n3}}/{2}} \right){\text{AD}}^{{{1}/{2}}} \nu^{{{1}/{2}}} {\text{C}} $$

where Ip is the peak current (A), n is the number of transferred electrons, A is the surface area (cm^2^), D is the diffusion coefficient (cm^2^/s), ν the potential scan rate (mV/s), and C is the concentration of reactants (mol/cm^3^). In order to investigate the active surface area of the electrodes, CV experiments were done using a 5 mM [Fe(CN)_6_]^3−^/[Fe(CN)_6_]^4−^ solution in aqueous 0.1 mol/L KCl at a potential scan rate range of 10–100 mV/s (Fig. 6a–d). The plot of the peak current vs. the square root of the scan rate helps us measure active surface area of the electrodes. The calculated active surface area for bare GCE, GCE/Bi_2_S_3_, GCE/rGO/Bi_2_S_3_, and GCE/rGO/PAMAM/Bi_2_S_3_ are 0.0464, 0.0580, 0.159, and 0.231 cm^2^, respectively. The results clearly show the significant effect of rGO and PAMAM/Bi_2_S_3_ nanomaterial in increasing the active surface area (up to five times with respect to bare GCE) of the electrode and, consequently its sensitivity.

### Electrochemical kinetic parameters investigation for sensor

The kinetic study of the proposed sensor was investigated using cyclic voltammetry analysis. Figure 6 represented cyclic voltammograms of 6.00 × 10^2^ nmol/L salbutamol in phosphate buffer (0.1 mol/L, pH = 5.0) on rGO, PAMAM/Bi_2_S_3_, GCE/rGO/PAMAM/Bi_2_S_3_, and bare GCE (5.00 × 10^2^ µmol/L salbutamol in PBS) at various scan rates. The results indicate that the oxidation current of salbutamol was linearly increased by increasing the scan rate. As shown in Fig. 6i, the peak currents are linearly related to the square root of the scan rate, which confirms that the oxidation process is diffusion-controlled. Also, the absence of any reduction peak for salbutamol indicates that the oxidation process is irreversible.

Laviron's equation expresses the relationship between Epa and v for irreversible electrochemical reaction^36^. Hence it is used to calculate the electron-transfer coefficient (α) and the rate constant of the electrochemical reaction (K_s_) (Eq. 3).

3 $$ {\text{Epa = E}}^{0} + { }\frac{RT}{{\left( {1 - \alpha } \right)nF{ }}}{\text{ln}}\frac{{\left( {1 - \alpha } \right)nF}}{RTKs} + { }\frac{RT}{{\left( {1 - \alpha } \right)nF}}{\text{ln v}} $$

where Epa, Ks, E^0^, n, ν, F, R, and T are anodic peak potential, surface heterogeneous electron transfer rate constant (s^−l^), formal redox potential, number of electrons transferred, scan rate (V/s), Faraday constant (96,485 C/mol), universal gas constant (8.314 J/K/mol) and temperature (298.15 K), respectively. Considering n = 1, the value of α was determined using the slope value of Epa vs. ln ν plot. The E^0^, which was obtained from the intercept of the plot, and α was applied in Eq. 4 to estimate Ks values at 0.05 Vs-1 (ΔE = Epa $-$ E^0^).

4 $$ {\text{Log}}\,{\text{Ks = }} \alpha {\text{log }}({1} - \alpha ) + \left( {1 - \alpha } \right){\text{log}}\alpha - \log \frac{RT}{{nF\upsilon }}\left( {\frac{nF\Delta E}{{2.3RT}}} \right) $$

The obtained Ks values are 0.0099 s^−1^ and 0.1294 s^−1^, and the obtained α values are 0.0036 and 0.0.6113 for bare GCE, GCE/ PAMAM/Bi_2_S_3_, GCE/rGO, and GCE/rGO/PAMAM/Bi_2_S_3_, respectively. The results indicate that through modifying the electrode surface, the electron transfer rate for salbutamol oxidation increases significantly.

### Figure of merit for the GCE/rGO/PAMAM/Bi_2_S_3_ sensor

#### Linearity and limit of detection

**Figure 7:**
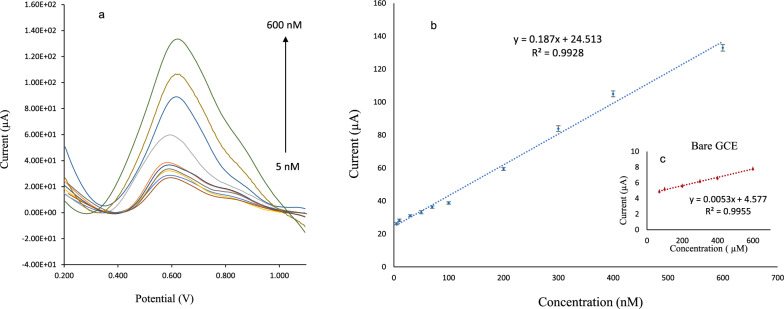
(**a**) DPV voltammograms of GCE/rGO/PAMAM/Bi_2_S_3_ sensor in 0.10 mol/L phosphate buffer (pH 5.0) containing different concentrations of SAL (5.00–6.00 × 10^2^ nmol/L). Plot of DPV current vs. concentrations of salbutamol (**b**) GCE/rGO/PAMAM/Bi_2_S_3_ (5.00–6.00 × 10^2^ nmol/L) and (**c**) GCE (7.00 × 10–6.00 × 10^2^ μmol/L).

The calibration curve was obtained using the peak current (µA) of SAL in DPV analysis. Different concentrations of SAL from 5.00 to 6.00 × 10^2^ nmol/L were examined under optimal conditions. The plot of ip vs. SAL concentrations is linear (y = 0.187x + 24.513, R^2^ = 0.9928) (Fig. 7). In order to compare the sensitivity of the GCE/rGO/PAMAM/Bi_2_S_3_ sensor to the bare GCE, the calibration curve was drawn for salbutamol (7.00 × 10–6.00 × 10^2^ µmol/L) using bare GCE. As shown in Fig. 7, the new nanocomposite has increased the sensitivity of the electrode about 35 times (from 0.005 to 0.187 µA µM^−1^ cm^−2^). The standard deviation for 5 replicated blank measurements was (0.0994); hence the limit of detection (LOD = 3sb/m) was 1.62 nmol/L, which is satisfactory.

### Selectivity

In order to evaluate the selectivity of the GCE/rGO/PAMAM/Bi_2_S_3_ sensor for salbutamol determination, the interference of common compounds such as ascorbic acid, urea, glucose, L-cysteine, starch, and two drugs, diltiazem, and dexamethasone were examined in two ratios of 1:10 and 1:5. The experimental result in the presence of 1:10 (salbutamol: L-cysteine, glucose, starch, diltiazem, and dexamethasone) and 1:5 (salbutamol: ascorbic acid, urea) had an error around or less than 5%. The resultant DPV responses shown in Fig. S4 indicate acceptable selectivity and no significant interference in GCE/rGO/PAMAM/Bi_2_S_3_ sensor performance for salbutamol determination.

### Repeatability and reproducibility

Repeatability and reproducibility are the essential features of an electrochemical sensor that demonstrates its practicality for real-time measurements. The repeatability of the generated sensor was determined by DPV intra-day (n = 5) and inter-day (n = 3) analyses. This study was performed at two different concentrations of SAL (5.00 × 10 and 3.00 × 10^2^ nmol/L). The relative standard deviation (RSD %) for the intra-day analysis were 2.61% and 1.52%, respectively. Moreover, the inter-day measurements RSDs were 2.85% and 3.50%, respectively. These results indicate that the precision of the GCE/rGO/PAMAM/Bi_2_S_3_ sensor is acceptable and it is suitable for SAL electrochemical measurements. In order to investigate the reproducibility, three dispersed modifier solutions were prepared at optimal values, and then the electrochemical response of the three modified electrodes was evaluated at 3.00 × 10^2^ nmol/L of SAL. The relative standard deviation of 1.88% indicates good reproducibility of the sensor. The long-term stability of the sensor was determined by measuring the signal of the sensor in a time interval of ten days, and calculating the ratio of the signal on different days to the signal of the fresh electrode. The modified electrode was stored in the refrigerator after preparation. Investigations showed that the sensor was well stable after ten days (Fig. S5).

### Real sample analysis

To characterize the performance and potential of the GCE/rGO/PAMAM/Bi_2_S_3_ sensor, detection of salbutamol in feed samples, milk, and sausage was carried out. Animal feed and poultry feed were treated as the real sample via the procedure reported by Noosang et al. ^37^. The extraction process is summarized as follows: 1.0 g of the feed dispensed in 5.0 mL of 0.20 mol/L phosphoric acid and methanol (1:4 v/v) was sonicated for 15 min, then centrifuged at 5000 rpm for 10 min. The supernatant was separated, and 1.0 mL of 0.1 mol/L HCl was added to the solution to remove proteins, followed by centrifugation at 5000 rpm for 10 min. The supernatant was dried at 60 °C, and the residue was then dissolved in 3.0 mL of PBS (0.1 mol/L, pH = 5.0) before electrochemical determinations. In order to evaluate the accuracy of measurements, various concentrations of SAL (3.00 × 10 and 10.0 × 10 nmol/L) were spiked into the real sample and followed by SAL determination in the sample. The milk, including various amounts of salbutamol, was analyzed. The milk sample was prepared by the method presented by Xia Niu et al. For this purpose; the milk was diluted 10 times in 0.1 mol/L phosphate solution (pH = 5.0), then centrifuged for 10 min at 10,000 rpm. The supernatant was used for analysis^38^. The preparation method for sausage samples was used with the following measures. 1 g of crushed sausage sample was kept in 30 mL 0.01 mol/L HCl at room temperature overnight. After removing precipitates, the supernatant, including the spiked certain amount of salbutamol, was used for electrochemical analysis^39^. As shown in Table 1, the obtained recoveries were satisfactory and indicate that the proposed sensor is suitable for salbutamol measurement in real samples.

**Table 1:** Analytical results of salbutamol in feed and food samples.

Sample	Salbutamol added (nmol/L)	Salbutamol Found (nmol/L)	RSD (%)	Recovery (%)
Animal feed	0.00	ND		–
	30.0	28.0 ± 0.981	3.5	93.4
	100.0	95.8 ± 3.07	3.2	95.8
Poultry feed	0.00	ND		–
	30.0	28.26 ± 1.02	3.6	94.2
	100.0	97.8 ± 3.33	3.4	97.8
Milk	0.00	ND		–
	30.0	26.55 ± 1.29	4.87	88.5
	100.0	91.2 ± 4.24	4.65	91.2
Sausage	0.00	ND		–
	30.0	26.85 ± 0.680	2.55	89.50
	100.0	84.06 ± 1.96	2.34	84.06

*ND* not detected.

### Comparing with other works

Analytical parameters of SAL determination using the GCE/rGO/PAMAM/Bi_2_S_3_ sensor were compared with previously reported works (Table 2). The proposed sensor has a linear concentration range up to two orders of magnitude, which is quite satisfactory and superior to others. The LOD is lower (better) or almost similar to those reported by others. Also, the precision (RSD) of our sensor is superior to others. Therefore, the generated sensor, is cost-effective and reliable in its possessing potentials. In addition, it has an acceptable linear range and high precision. Therefore, it is proposed for SAL determination due to its good performance.

**Table 2:** Comparison of the present approach with other reported methods for detecting salbutamol.

Modifier	Electrode	Method	Linear range	LOD	RSD (%)	References
Graphene oxide and poly(O-nitrobenzoic acid)	GCE	DPV	64.23–626.2 µmol/L	56.00 µmol/L	6.2	^9^
Nano-Au/L-cys/MWCNTs NF	GCE	LSV	0.09–7.00 µmol/L	0.05 µmol/L	4.3	^10^
Polytaurine/ZrO2	GCE	LSV	5–220 µmol/L	0.02 µmol/L	4.9	^40^
Poly(AHNSA)	GCE	DPV	0.2–8 µmol/L	0.068 µmol/L	–	^41^
GP-PEDOT: PSS	SPCE	CV	5–550 µmol/L	1.25 µmol/L	–	^8^
Bi2Te3/GCN	SPCE	DPV	0.01–892.5 µmol/L	1.36 nmol/L	2.96	^42^
WS2/AC	GCE	DPV	1–210 µmol/L	0.52 µmol/L	–	^43^
hafnium-doped tungsten oxide (Hf.WO3) nanorods	CPE	SWV	9–500 nmol/L	2.42 nmol/L	–	^44^
Ag–N-rGO-MIP	GCE	DPV	0.03–20.00 µmol/L	7 nmol/L	4.83	^1^
rGO-PAMAM-Bi_2_S_3_	GCE	DPV	5.00–6.00 × 10^2^ nmol/L	1.62 nmol/L	2.61, 1.52	Present work

*MWCNT* Multi-walled carbon nanotubes; *SPC* screen printed carbon electrode; *GP-PEDOT: PSS* graphene-poly(3, 4-ethylenedioxythiophene): poly(styrene-sulfonate); *NF* Nafion; *AHNSA* 4-Amino-3-hydroxynaphthalene sulfonic acid; *GCN* graphitic carbon nitrides sheets; *AC* activated carbon; *CPE* carbon paste electrode; *SWV* square wave voltammetric method; *MIP* molecularly imprinted polymer.

## Materials and methods

### Chemicals and materials

Ethylenediamine (EDA), methyl acrylate, graphite powder, sodium nitrate, sulfuric acid (95%), potassium permanganate, hydrogen peroxide (30%), hydrochloric acid, thioacetamide, bismuth (III) nitrate pentahydrate, nitric acid, dimethylformamide (99%), acetic acid (99.5%), sodium hydroxide (96%), aluminium oxide (5 µm), methanol, potassium chloride, phosphoric acid (85%), boric acid (99.5%), K_4_[Fe(CN)_6_]·3H_2_O, and K_2_[Fe(CN)_6_] were supplied from Merck company (Darmstadt, Germany). Salbutamol was supplied by Darou-Pakhsh pharmaceutical company (Tehran, Iran). Animal and poultry feed products were purchased from Tavanmehr livestock and poultry feed factory (Kerman, Iran). Milk and sausages were purchased from a local grocery store.

### Instruments and software

Voltammetric measurements were taken using PalmSens3 electrochemical apparatus supplied with PSTrace 4.8 software (PalmSens Instrument BV, Houten, Netherlands). The instrument has a three electrodes system containing a modified GCE/rGO/PAMAM/Bi_2_S_3_ (working electrode), a Pt electrode (counter electrode) and a 3.0 mol/L Ag/AgCl (reference) electrode. Adjustments of pH were accomplished by a digital Bp3001 pH meter (Trans Instruments, Singapore). Ultrasonic bath RK-255-H (Bandelin Electronic. Co, Berlin, Germany) was utilized for dispersing and homogenizing the constituents of mixtures. Freeze dryer LYOQUEST-85 (Telstar, Spain) was used to dry the synthesized rGO. The synthesized Bi2S3 was dried in the vacuum oven FTVO-702 (Sci Finetech, Seoul, Korea). Morphology of nanocomposite was specified using field emission scanning electron microscopy (FE-SEM) (sigma VP, ZEISS, Germany), EDX (Oxford Instruments plc, Tubney Woods, Abingdon, UK), FT-IR (TENSOR 27, Brucker, Germany) instruments. The Design-Expert software version 11.0 was applied for controlling experimental conditions, response surface modeling, and optimization.

### Preparation of modifier components (rGO/PAMAM/Bi_2_S_3_)

The optimum amounts of rGO, PAMAM, and Bi_2_S_3_ were used as modifiers to improve the electrochemical performance of the sensor. In the following sections, their synthesis methods are described.

### Synthesis of GO and rGO

Modified Hummer’s method was used to synthesize GO^45^. Briefly, 1.0 g of graphite powder and 0.50 g of NaNO_3_ were mixed in 23.0 mL of 95% H2_S_O_4_ placed on an ice bath and stirred for 30 min. Then, 3.0 g of KMnO4 was added gradually to prevent the large temperature rise. The mixture was stirred overnight at 35 °C. followed by the stirring procedure, 60.0 mL D.I. water was added, and the mixture was stirred again at 35 °C for 14 h. After decline of temperature to a cooling level of room temperature, 500 mL of D.I. water was added, followed by 7.00 mL of 30% hydrogen peroxide (H_2_O_2_). The precipitate was separated and washed with 1.0 mol/L HCl solution and then the washing process continued with ultrapure water several times. GO powders are obtained after drying the final product under vacuum at 50 °C for 6 h.

The reduced graphene oxide was synthesized according to the reported work of Xiaoyi Yan et al.^21^. The synthesis procedure was as follows: 30.0 mL aqueous solution including 15 mg GO and 12 mmol/L of thioacetamide (TAA) was transferred into a 40 mL Teflon-lined stainless-steel autoclave. The reaction was completed after 8 h at 160 °C. Finally, the obtained rGO was centrifuged, washed with ultrapure water, and dried in a vacuum freeze drier.

### Synthesis of Bi_2_S_3_

The Bi_2_S_3_ was synthesized using the method proposed by Yang Zhao et al.^46^. In this method, Bi(NO_3_)_3_ was sonochemically hydrolyzed in the presence of TAA. In order to implement this method, 75 mg TAA was dispersed in 40.0 mL distilled water by sonication. Then, 5.00 mL of 0.40 mol/L HNO_3_ solution containing 0.243 g Bi(NO_3_)_3_.5H_2_O was gradually added to the TAA mixture, and the resulting suspension was stirred for one hour. Next, the precipitate was dispersed in 20.0 mL DMF and transferred into a stainless-steel autoclave for 2 h at 150 °C. Finally, the synthesized product was washed several times with distilled water and dried in a vacuum oven at 60 °C.

### Synthesis of PAMAM (G3)

The PAMAM dendrimers were synthesized using the reported method by Janek Peterson et al.^47^. Basically, the procedure contains two consecutive steps: synthesis of ester-terminated (half-generation) PAMAM dendrimers, and synthesis of amino-terminated (full-generation) PAMAM dendrimers. The Michael addition of a primary amine to methyl acrylate and amidation of the generated multiset was repeated several times to produce higher generations of PAMAM dendritic molecules (Fig. S6).

### Fabrication of the modified sensor

Before modification, the glassy carbon electrode (GCE) was polished with Al_2_O_3_ (5 µm) slurry supported on a patch of cloth, then it was rinsed with distilled water, and dried at room temperature. In order to prepare the modifier, the optimized values of the PAMAM and Bi_2_S_3_ were mixed in an ultrasonic bath for 30 min and then a certain amount of rGO was added to the PAMAM/Bi_2_S_3_ solution and mixed in an ultrasonic bath for 30 min. The optimum values for modifiers were obtained using the experimental design method. Then, 8 µL of the dispersed rGO/PAMAM/Bi_2_S_3_ solution was dropped on the surface of GCE. The GCE/ rGO/PAMAM/Bi_2_S_3_ was dried in an oven at 40 °C. For the sake of comparison, the GCE /rGO and GCE/Bi_2_S_3_ were prepared similarly. The modified electrodes were used for electrochemical measurements.

### Electrochemical determination of Salbutamol

The electrochemical determination of salbutamol was done using the differential pulse voltammetry method (DPV) as a highly sensitive electrochemical method. The different parameters of the differential pulse voltammetry method were optimized for salbutamol measurements, and all measurements were performed under optimal conditions. For pre-treatment, the potential of 0.50 V was applied to the electrode for 300 s under stirring. After 5 s of equilibrium time, the potential range + 0.20 to + 1.10 V (vs. Ag/AgCl) was applied. The pulse amplitude, pulse time, and scan rate were 0.30 V, 5 ms, and 0.03 V/s, respectively. Various concentrations of salbutamol were provided in 0.1 mol/L phosphate buffer (pH = 5.0) as the supporting electrolyte.

## Conclusion

This work presents a novel dendrimer-based nanocomposite for the fabrication of the sensor in the determination of salbutamol. The combination of rGO, PAMAM, and Bi_2_S_3_ nanomaterials in constructing the sensor was used. The most appropriate composition ratio of nanocomposite components for sensor fabrication with taking into account the influence of interactions among these parameters was determined using the RSM. Using RSM, the optimum conditions for these factors were theoretically predicted and this was successfully approved through experimental tests. According to the dendrimer structure of PAMAM, it was used in the preparation of the nanocomposite in order to increase the active surface area of the sensor. Experimental evidence shows the active surface area of the modified sensor (0.2310 cm^2^) is five times that of the bare GCE (0.0464 cm^2^). The presence of agonists such as salbutamol in animal and poultry feed can cause acute poisoning in humans, with symptoms of cardiac palpitation, vomiting, headache, muscular tremor, muscular pain, nervousness, dizziness, chills, fever, and nausea. Therefore, the development of a sensor for fast, sensitive and accurate determination of this drug is very important. This sensor has a linear range, RSD, LOD, and sensitivity 5.00–6.00 × 10^2^ nmol/L, 2.61% and 1.52%, 1.62 nmol/L, and 0.187 µA µM^−1^ cm^−2^, respectively which is compatible with or better than the other methods. Application of the proposed sensor for real sample analysis indicates that it is well suited (highly accurate and precise) for determination of salbutamol in livestock and poultry feed, milk, and sausage sample. The complexity of meat product samples, and subsequently, the more difficult analyte preparation and extraction process, can affect the sensor response in these real samples. Therefore, the slight difference in the performance of the sensor in the sausage sample can be attributed to the complexity of the meat products.

## Supplementary Information

Supplementary Information.

## Data Availability

The datasets generated and analyzed during the current study are available via this link: https://drive.google.com/file/d/1h3A0r5_cU5-xDeQ4O8DjVKon-O23B48K/view?usp=share_link.
