# Supplementary material for: Development of bismuth sulfide nanorods and polyamidoamine dendrimer on reduced graphene oxide as electrode nanomaterials for electrochemical determination of salbutamol
Source: Sci Rep. 2023 Jun 1;13:8902. doi: 10.1038/s41598-023-36028-0 (PMC10235058; doi:10.1038/s41598-023-36028-0)
Supplement: Supplementary file 1 — Supplementary Information. [file 41598_2023_36028_MOESM1_ESM.pdf]

Development of bismuth sulfide nanorods and polyamidoamine dendrimer on reduced graphene oxide as electrode nanomaterials for electrochemical determination of salbutamol

*Mahshid Padash[a,b], Shahab Maghsoudi[a, *], and Mehdi Mousavi[a]*

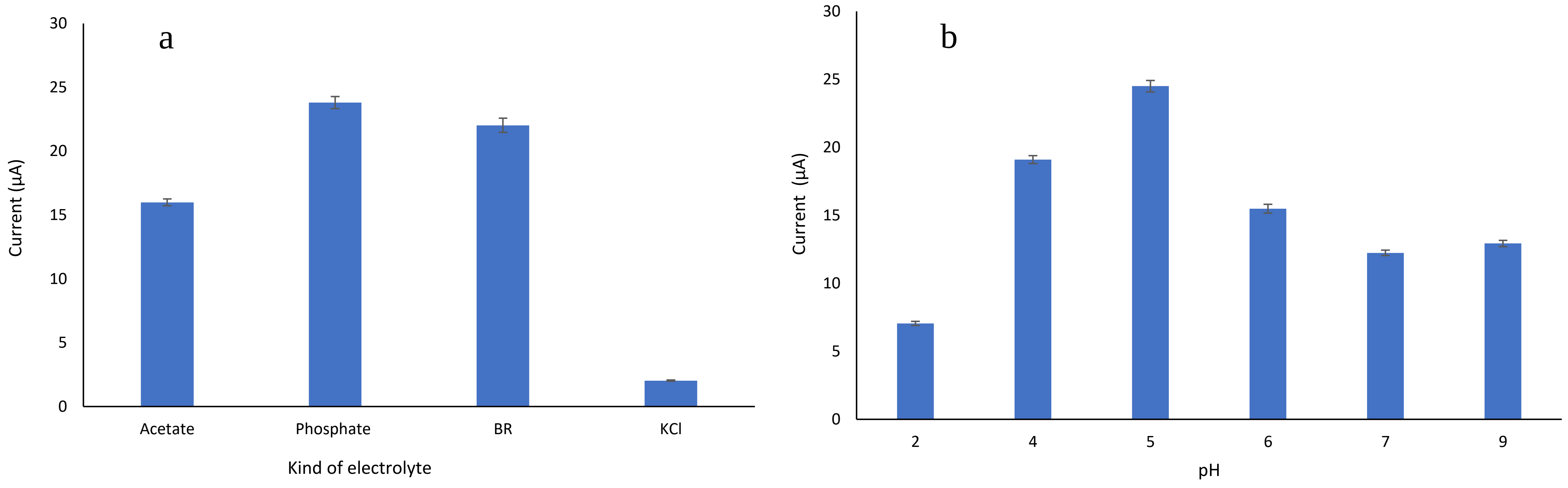

Figure S1. Effect of (a) kind of electrolyte, (b) different pH of 0.1 mol/L phosphate solution (4.0-7.0)

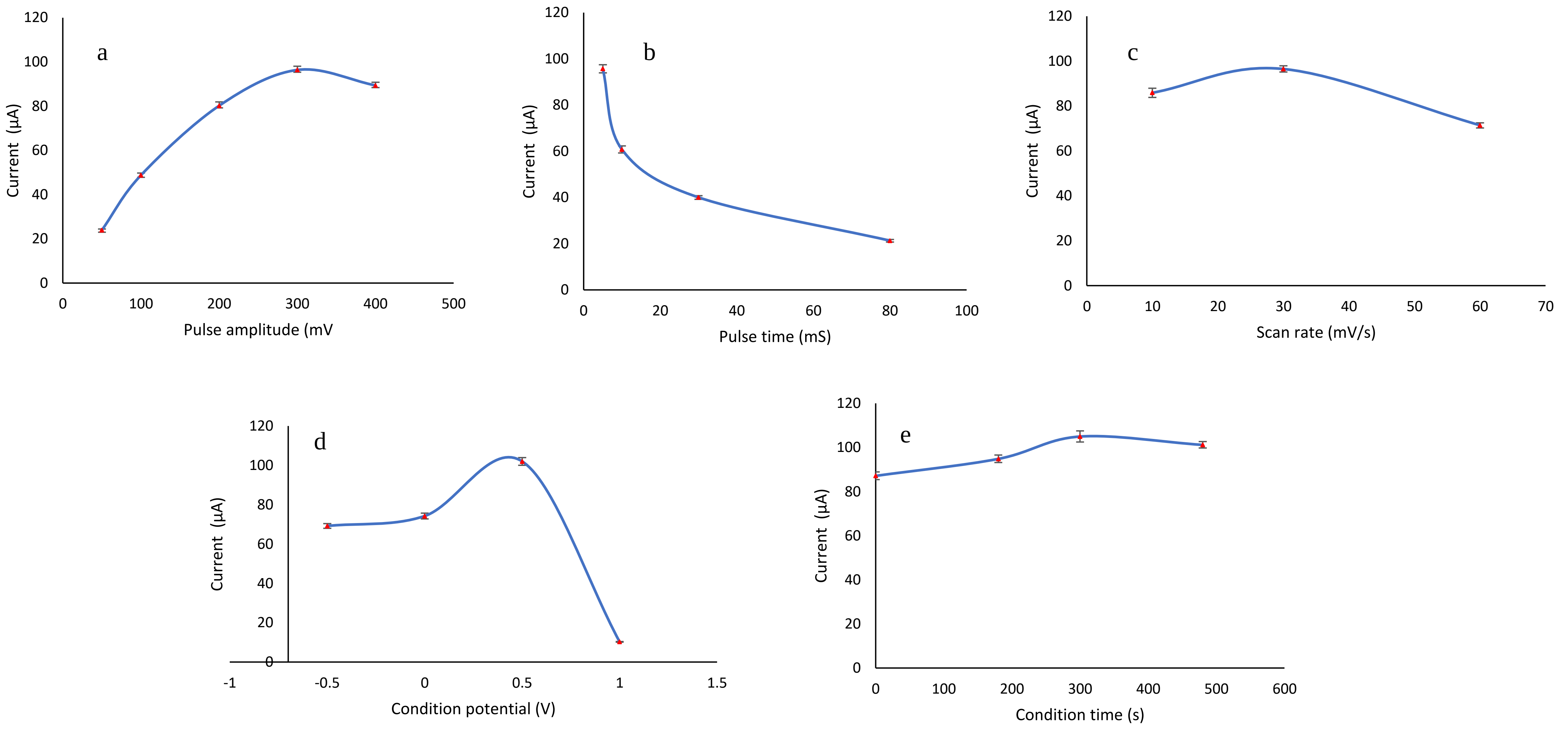

Figure S2. The effect of (a) pulse amplitude, (b) pulse time, (c) scan rate, (d) condition potential, and (e) condition time on DPV current for detection of 4.0×10^2 nmol/L SAL in 0.1 mol/L phosphate buffer (pH=5.0).

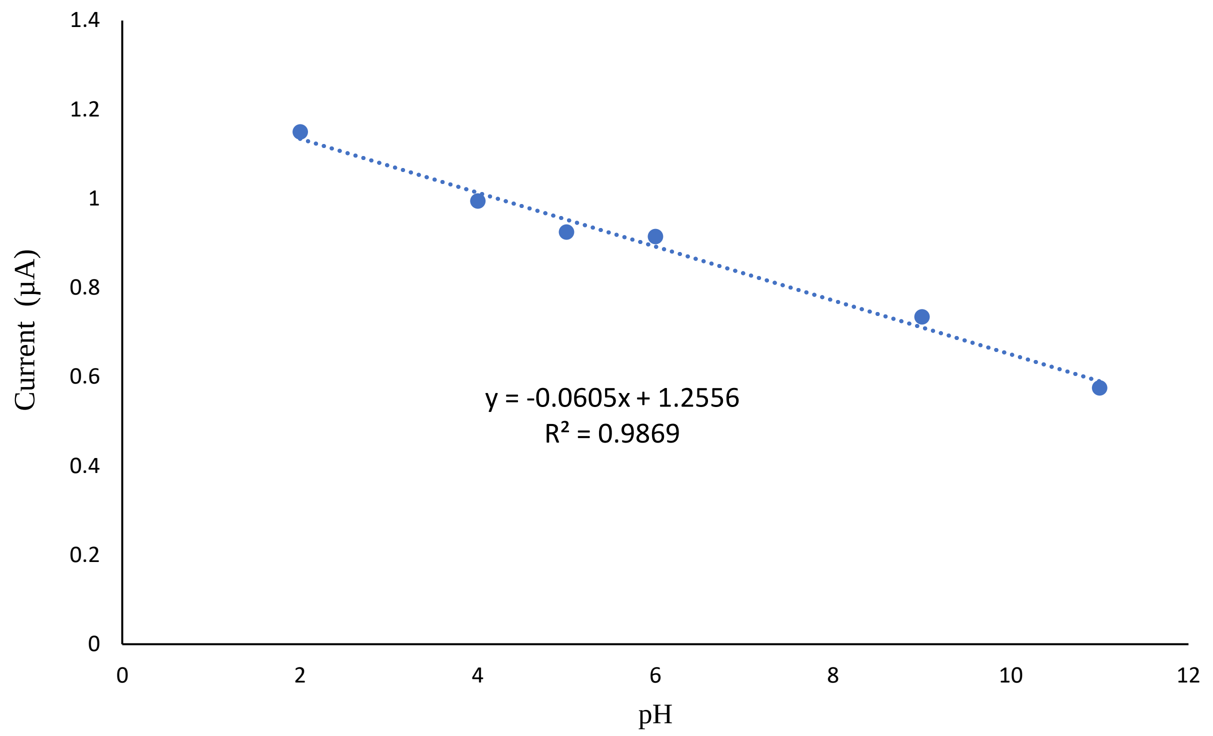

Figure S3. The plot of the potential of the cathodic peak in CV voltammogram vs. pH

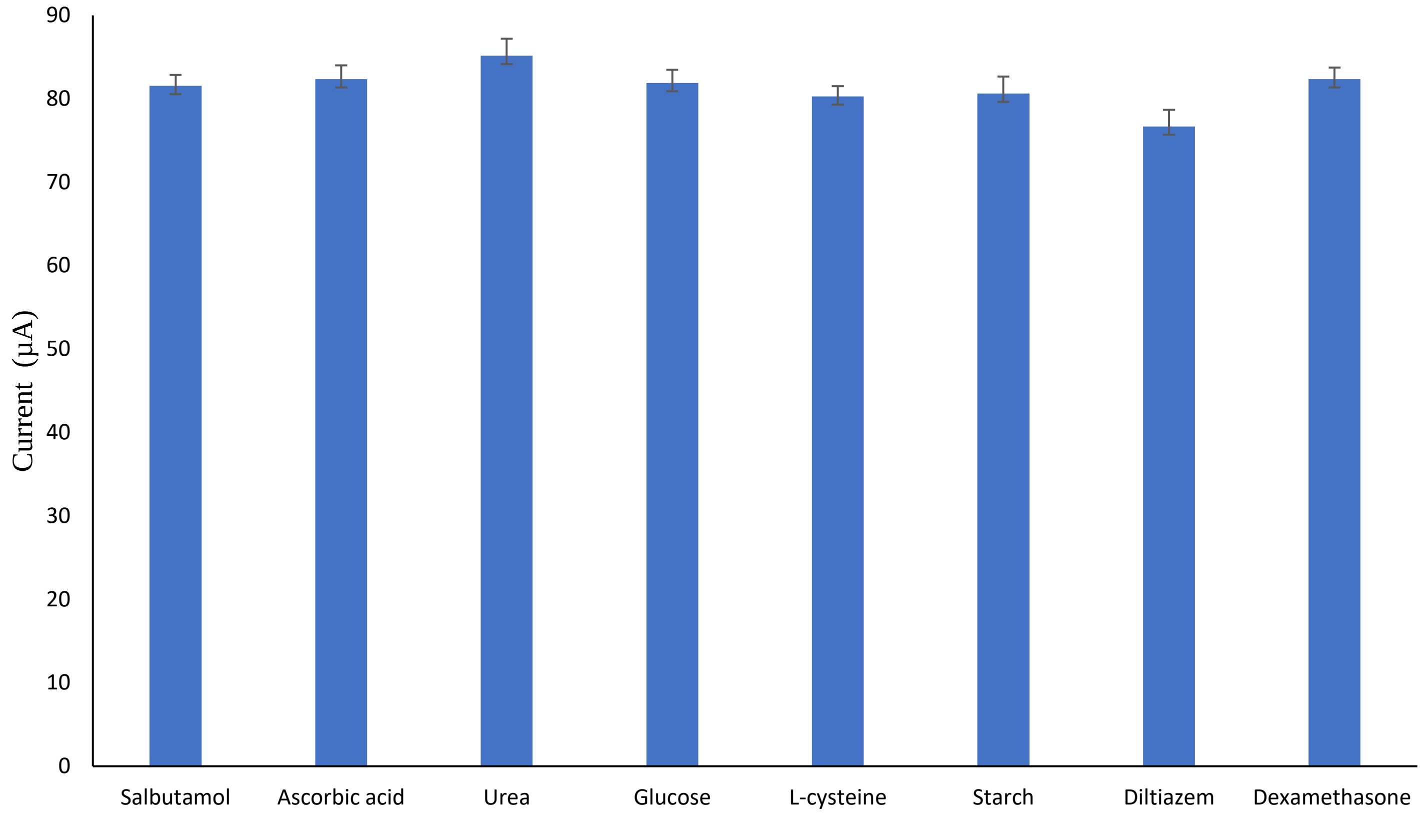

Figure S4. Evaluation of the selectivity of the proposed sensor for determination of salbutamol (300 nM) in the presence of other substances

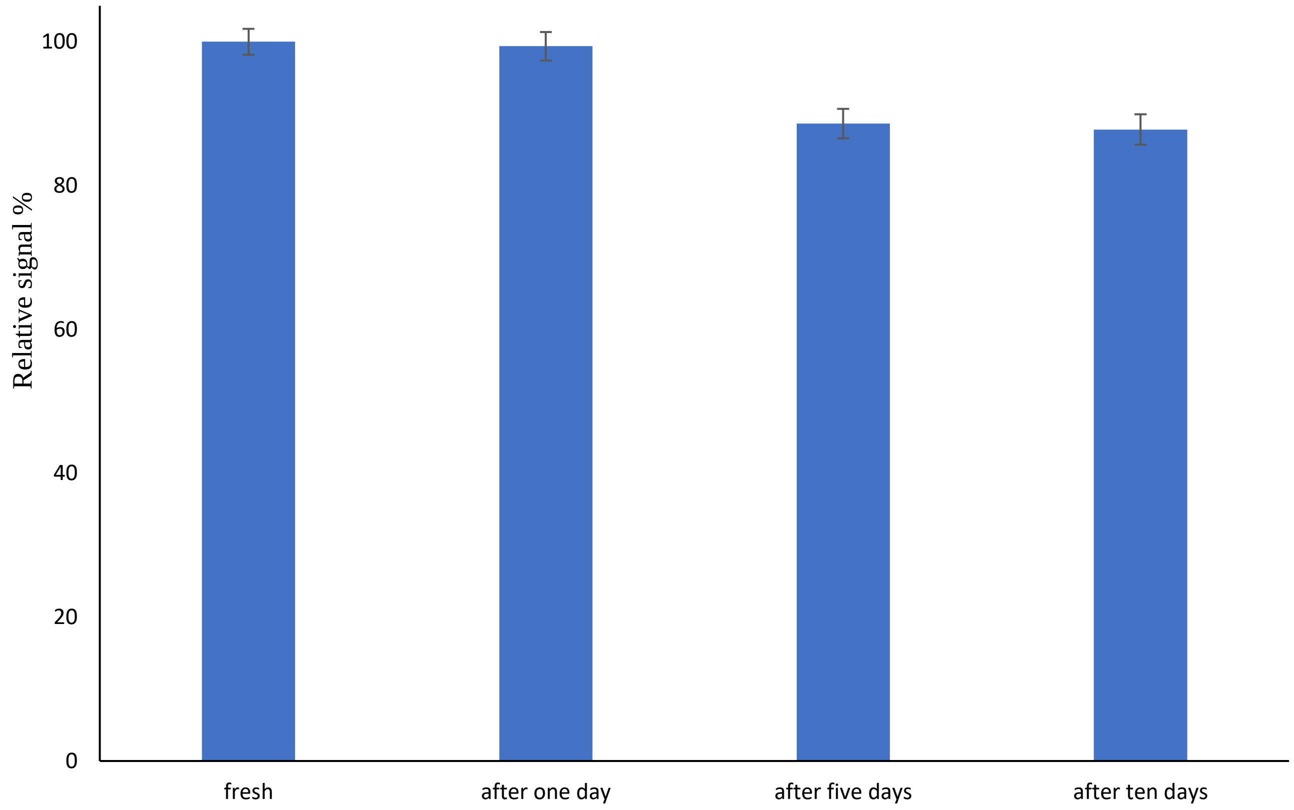

Figure S5. The stability of the modified electrode after one, five and ten days for salbutamol determination (200 nM in PBS 0.1M, pH=5.0)

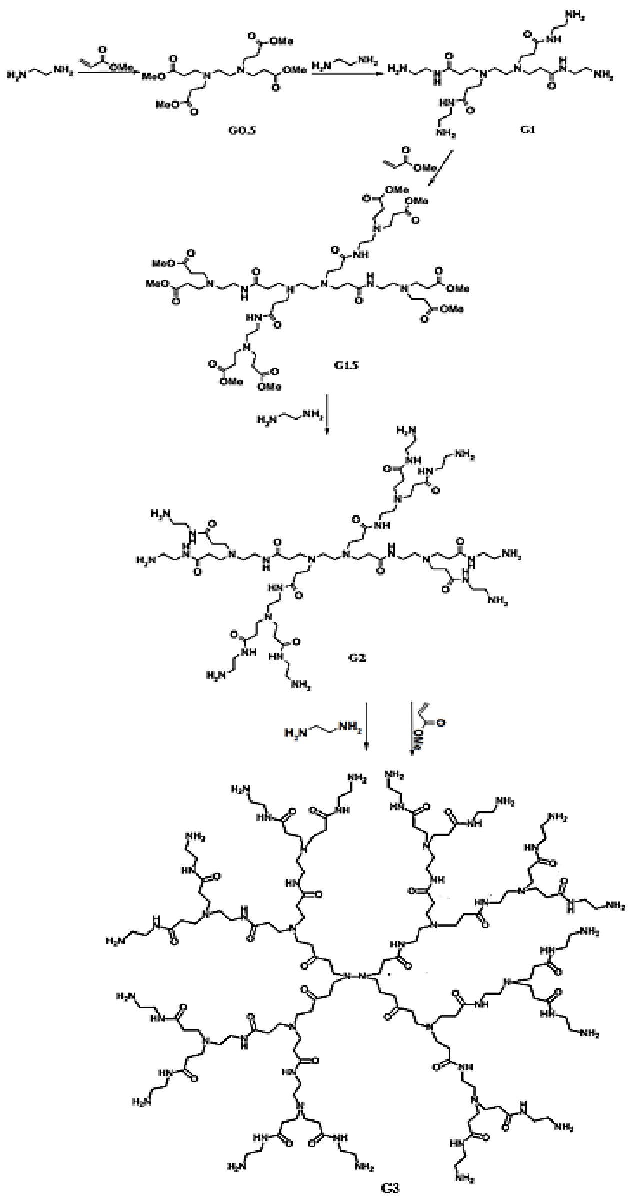

Figure S6. Diagram of the synthesis of EDA-core PAMAM dendrimer

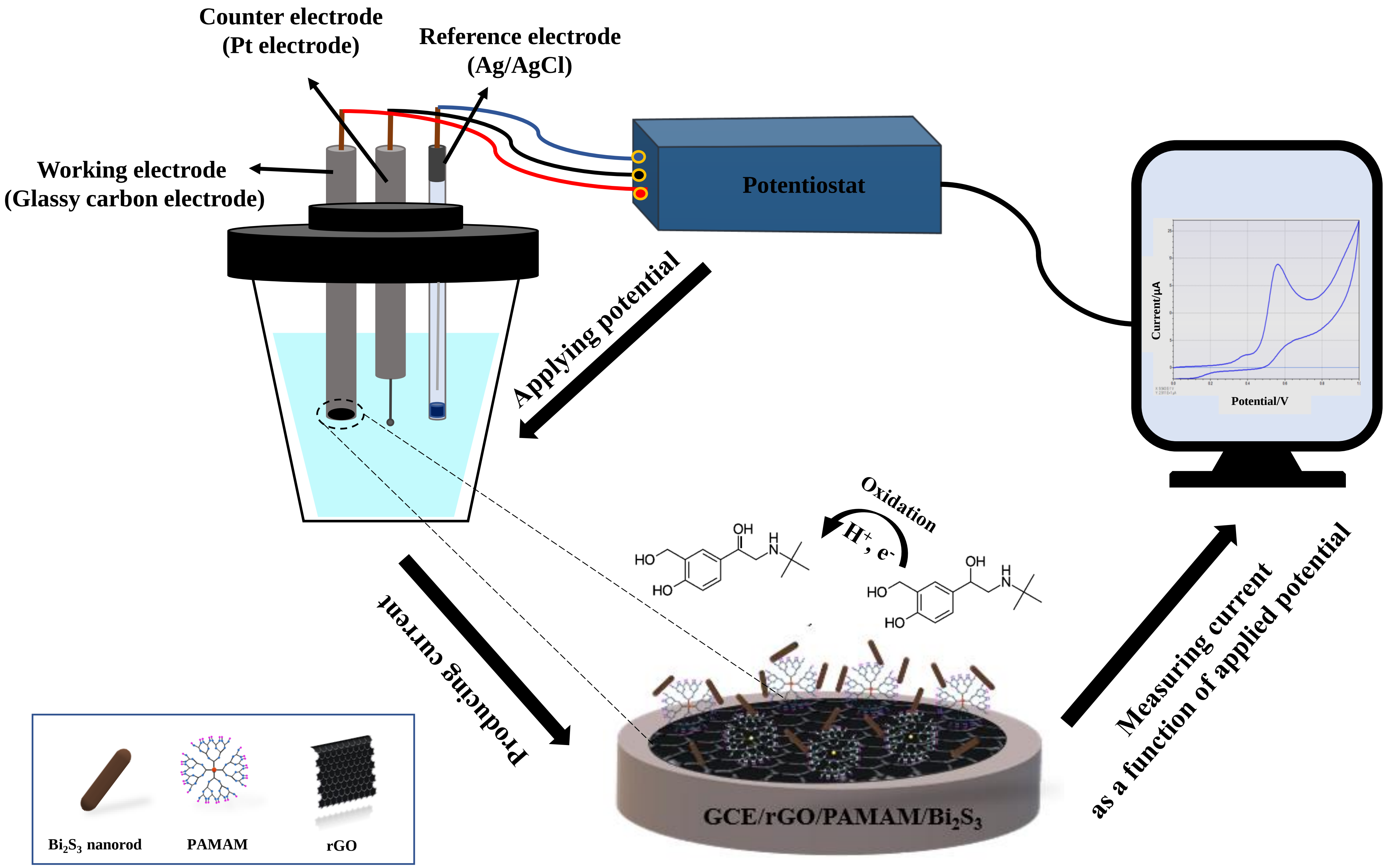

Figure S7: Schematic of sensing phenomena

Table S1.Experimental factors and levels in CCD

Factor	Name	Units	Coded and Uncoded Factors levels				
			(-α)	(-1)	(0)	(+1)	(+α)
A	Bi_2S_3	mg/mL	0.104	0.800	1.900	3.000	3.700
B	rGO	mg/mL	0.010	0.200	0.500	0.800	0.989
C	PAMAM	mg/mL	0.073	0.200	0.400	0.600	0.727
